# Dataset for quantum-mechanical exploration of conformers and solvent effects in large drug-like molecules

**DOI:** 10.1038/s41597-024-03521-8

**Published:** 2024-07-07

**Authors:** Leonardo Medrano Sandonas, Dries Van Rompaey, Alessio Fallani, Mathias Hilfiker, David Hahn, Laura Perez-Benito, Jonas Verhoeven, Gary Tresadern, Joerg Kurt Wegner, Hugo Ceulemans, Alexandre Tkatchenko

**Affiliations:** 1 Department of Physics and Materials Science, University of Luxembourg, L-1511 Luxembourg City, Luxembourg; 2 Institute for Materials Science and Max Bergmann Center of Biomaterials, TU Dresden, 01062 Dresden, Germany; 3 Drug Discovery Data Sciences (D3S), Janssen Pharmaceutica NV, Turnhoutseweg 30, 2340 Beerse, Belgium; 4 Computational Chemistry, Janssen Pharmaceutica NV, Turnhoutseweg 30, 2340 Beerse, Belgium; 5 Drug Discovery Data Sciences (D3S), Johnson & Johnson Innovative Medicine, 301 Binney Street, MA 02142 Cambridge, USA

**Keywords:** Computational chemistry, Chemical physics, Quantum mechanics, Chemical physics

## Abstract

We here introduce the Aquamarine (AQM) dataset, an extensive quantum-mechanical (QM) dataset that contains the structural and electronic information of 59,783 low-and high-energy conformers of 1,653 molecules with a total number of atoms ranging from 2 to 92 (mean: 50.9), and containing up to 54 (mean: 28.2) non-hydrogen atoms. To gain insights into the solvent effects as well as collective dispersion interactions for drug-like molecules, we have performed QM calculations supplemented with a treatment of many-body dispersion (MBD) interactions of structures and properties in the gas phase and implicit water. Thus, AQM contains over 40 global and local physicochemical properties (including ground-state and response properties) per conformer computed at the tightly converged PBE0+MBD level of theory for gas-phase molecules, whereas PBE0+MBD with the modified Poisson-Boltzmann (MPB) model of water was used for solvated molecules. By addressing both molecule-solvent and dispersion interactions, AQM dataset can serve as a challenging benchmark for state-of-the-art machine learning methods for property modeling and *de novo* generation of large (solvated) molecules with pharmaceutical and biological relevance.

## Background & Summary

### Introduction

In pharmaceutical research and development, computational chemistry can play an integral role in expediting candidate drugs into the clinic. Particularly, quantum-mechanical (QM) methods (*e.g*., density-functional theory (DFT), post-Hartree-Fock approaches, and quantum Monte Carlo) have been utilized to describe covalent and non-covalent interatomic interactions and to estimate diverse physicochemical properties of molecular systems^1,2^. QM methods can for instance be used to understand the reactivity of covalent binders^3,4^, evaluate conformational energy landscapes of ligands^5^, study the stability of potential active pharmaceutical ingredients, calculate theoretical acidity constants^6^, or calculating theoretical charges to more accurately capture electrostatic properties and surfaces. However, the computational cost and the challenge of conducting QM calculations at a large scale present a limitation to their widespread use in drug discovery pipelines. Scanning conformational landscapes through QM calculations performed at DFT levels of theory typically takes several hours for a single ligand of typical pharmaceutical size (*e.g*., 30-40 heavy atoms) on a single computer. As these calculations are readily parallelizable, supercomputers can be employed to enhance throughput enabling the screening of a few hundred compounds, but it remains challenging to perform these calculations at the scale of virtual libraries which can easily be composed of tens of thousands of compounds.

Accelerated QM methods have emerged as promising solutions in recent years, offering a balance between accuracy and computational efficiency. These can take the form of quantum fragmentation methods^7,8^, semi-empirical methods (*e.g*., parametric method series^9^, density functional tight-binding (DFTB)^10,11^ or its extended version (GFNn-xTB)^12,13^) as well as machine learning (ML) models^14–20^ capable of optimizing geometries or estimating physicochemical properties. The resulting acceleration enables researchers to include QM-based knowledge as a part of their workflow. Accordingly, relevant QM datasets of small organic molecules have widely assisted the development of ML-based approaches for a fast and accurate estimation of structural, vibrational, and electronic properties of complex organic molecules^21–24^. Among them, one can find QM7^25–27^, QM9^28,29^, QM7-X^30^, MD17^15,31^, MD22^32^, ANI-1^14,33^, ANI-1x/ANI-1ccx^34^ and AIMNet-NSE^35^. While these QM datasets have significantly advanced the field of computational chemistry, they do exhibit certain limitations that stem from three important facts. First, they primarily consist of molecules that are considerably smaller than what is commonly encountered in modern medicinal chemistry. Second, their structures have been optimized using theoretical models that do not account for molecule-solvent and collective dispersion interactions. Lastly, they have not fully explored the vast conformational landscape inherent in these molecules. Especially, the interaction between the molecule and the chemical environment (*i.e*., solvent) is crucial when investigating molecules of pharmaceutically relevant size, as it is well-known that drug binding does not occur *in vacuo* but rather *in solutio*^36^. Indeed, there is extensive literature discussing the solvent effects on the properties of specific molecular systems^37–41^. For instance, Gorges *et al*.^38^ found that solvation can have a substantial effect of several cal/mol ⋅ K on the entropy of 25 commercially available drug molecules as a result of large conformational changes. The geometry, energetics, HOMO/LUMO energies, dipole moment, and polarizability of formaldehyde and thioformaldehyde have also been reported to change upon solvation in solvents of low polarity^39^. Similarly, the molecule-solvent interaction can affect the dynamic stability and antiviral inhibitory potential of Cissampeline^40^ as well as the chemical reaction type S_N_2 between Cl^−^ and CH_3_Cl^41^. Omitting solvent effects can thus result in inappropriate treatment of conformations, tautomers, physicochemical properties, or molecular reactivity. In the computational modeling of molecular systems, solvents can either be considered implicitly or represented explicitly. However, owing to the computational cost and intricate nature of explicitly representing the solvent, most QM studies opt for the utilization of implicit solvent models such as conductor-like screening model for real solvents (COSMO-RS)^42^, modified Poisson-Boltzmann (MPB)^43^, and Generalized Born (GB)^44^ model augmented with the hydrophobic solvent accessible surface area term (GBSA)^45^.

Lately, to overcome the molecular size limitation within benchmark QM datasets, several efforts have been made to generate datasets that comprehensively explore the conformational space of large and flexible molecules along with their associated QM properties calculated in gas phase or solvent, see Table 1. For instance, the QMugs^46^ collection comprises 19 QM properties of circa 2 M gas-phase conformers of 665, 911 molecules with up to 100 non-hydrogen atoms computed using *ω*B97X-D^47^ density functional and the def2-SVP basis set. The OE62^48^ dataset covers 61, 489 molecules with up to 92 non-hydrogen atoms that were optimized in gas phase using PBE(tight) level of theory supplemented with Tkatchenko-Scheffler van der Waals (TS) interaction^49^. This dataset also contains 3 QM properties for 30, 876 structures evaluated using PBE0(tight) level of theory together with implicit water defined by the Multipole Expansion (MPE) model^50^. Regarding the vast GEOM collection (which stands for Geometric Ensemble Of Molecules)^51^, only 1.3 M conformers corresponding to 1, 511 BACE^52^ molecules were generated considering molecule-solvent interactions described by the analytical linearized Poisson-Boltzmann (ALPB)^53^ model of water. From here, 455, 000 conformers of 534 BACE molecules were selected and used for further geometry optimization calculations using r2scan-3c functional with C-PCM^54^ (which stands for conductor-like polarizable continuum model) implicit model of water and, posteriorly, 6 QM properties were collected. Moreover, Eastman *et al*.^55^ have recently introduced the SPICE dataset (which is short for Small-molecule/Protein Interaction Chemical Energies) that explicitly considers the interaction between the molecule and water molecules *via* Amber14 classical force field to get 1, 300 (equilibrium and non-equilibrium) structures of 26 amino acids. Energies, atomic forces, and other 6 QM properties were computed using the *ω*B97M-D3(BJ) functional and def2-TZVPPD basis set. Despite these efforts, challenges remain to enable a better understanding of solvent effects as well as collective dispersion interactions in the chemical space of large drug-like molecules, including: (i) assessing the accuracy and reliability of QM structures and properties with respect to the employed density-functional approximation, especially for larger and more flexible molecules in which van der Waals (vdW) and molecule-solvent interactions are stronger, (ii) offering a large set of molecular (global) and atom-in-a-molecule (local) physicochemical properties that would enable a comprehensive exploration of these interactions in structure-property and property-property relationships throughout chemical space, and (iii) providing accurate and reliable QM data that will enable the construction of models for describing covalent and non-covalent vdW interactions in large (solvated) molecules.

**Table 1:** Main characteristics of current quantum-mechanical datasets of large-sized molecular systems.

Dataset	Total structures	Total molecules	Max. total atoms	Max. heavy atoms	Elements	Geometry level of theory	Solvent model	Property level of theory	Solvent model	Total DFT properties
QMugs^46^	1, 992, 984	665, 911	228	100	10	GFN2-xTB	—	*ω*B97X-D/def2-SVP	—	19
OE62^48^	30, 876	30, 876	174	92	16	PBE(tight)+TS	—	PBE0(tight)	MPE	3
BACE^51^	455, 000	534	115	61	9	r2scan-3c/mTZVPP	C-PCM	r2scan-3c/mTZVPP	C-PCM	6
Amino acids^55^	1, 300	26	96	39	5	Amber14 FF	explicit	*ω*B97M-D3(BJ) /def2-TZVPPD	—	10
AQM-gas	59, 783	1, 653	92	54	8	DFTB3+MBD	—	PBE0(tight)+MBD	—	36
AQM-sol	59, 783	1, 653	92	54	8	DFTB3+MBD	GBSA	PBE0(tight)+MBD	MPB	40

We have selected datasets where molecule-solvent and/or van der Waals interactions have been considered during their generation procedure. The BACE and Amino acids sets have been extracted from the GEOM and SPICE collections.

**Fig. 1:**
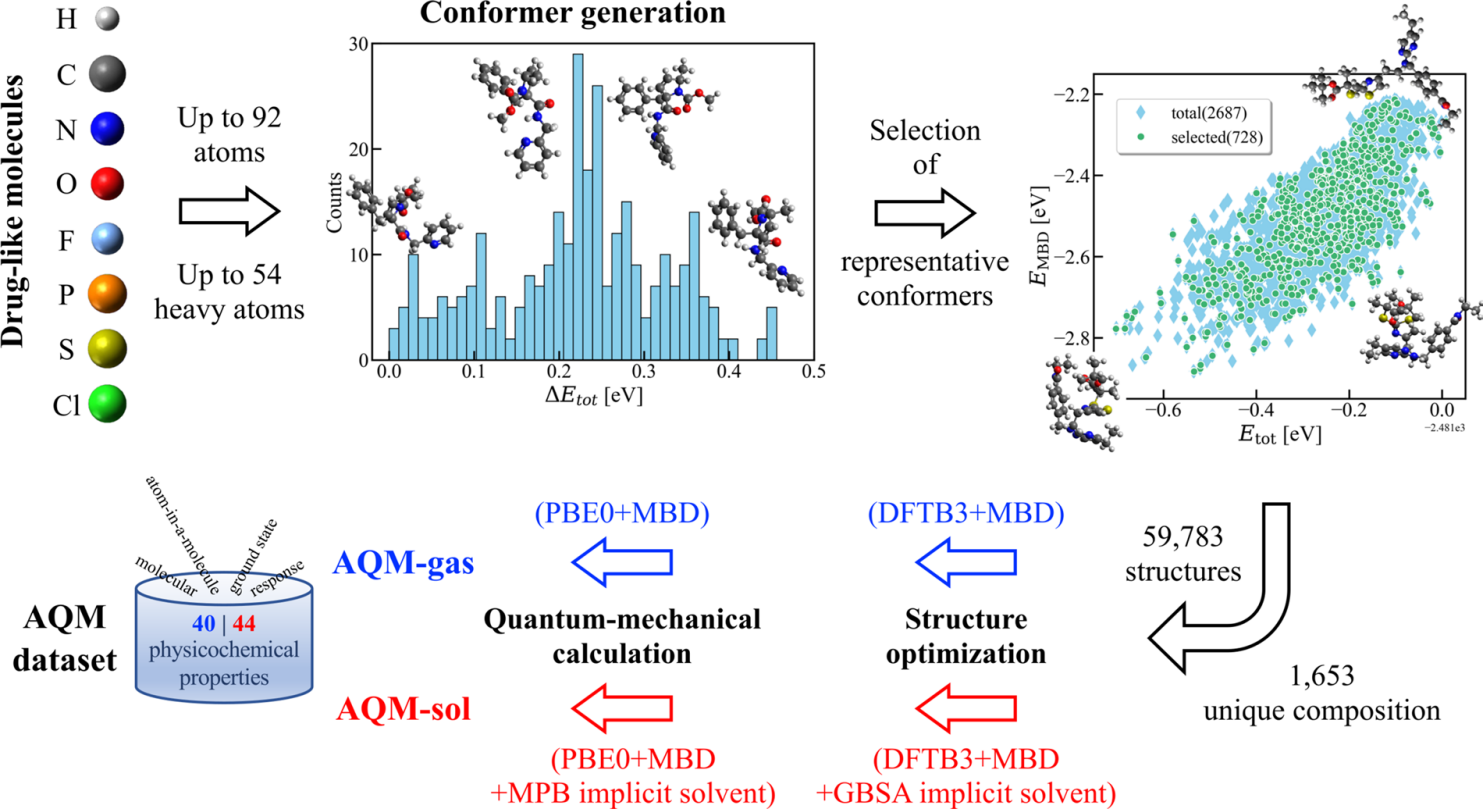
Scheme of the generation procedure of Aquamarine (AQM) dataset. We have here investigated molecules containing C, N, O, H, Cl, S, P, and F atoms. Then, we generated the conformers per molecule using the conformational search workflow implemented in CREST code which employs a metadynamics sampling with an additional genetic z-matrix crossing step. A set of representative conformers is selected using an energetic analysis considering the DFTB total energy *E*_tot_ and many-body dispersion (MBD) energy *E*_MBD_, see “Methods”. Thus, the current version of AQM dataset contains 59, 783 low-and high-energy conformers of 1, 653 molecules up to 54 non-hydrogen atoms (mean: 28.2) with a total number of atoms ranging from 2 to 92 (mean: 50.9). All structures in the final set of conformers are optimized using DFTB3+MBD and DFTB3+MBD supplemented with the GBSA implicit solvent model of water. Afterward, over 40 molecular (global) and atom-in-a-molecule (local) QM properties of gas-phase and solvated structures are computed by performing single point calculations at the PBE0(tight)+MBD level of theory and further enhanced with the MPB implicit solvent model of water, respectively. Hence, we have two different AQM subsets, namely AQM-gas and AQM-sol.

In this work, we introduce the Aquamarine (AQM) dataset with the aim of addressing these challenges. The current version of AQM contains an extensive conformational sampling of 1, 653 molecules with up to 54 (mean: 28.2) non-hydrogen atoms (including C, N, O, F, P, S, and Cl), producing a total of 59, 783 low-and high-energy conformers with a total number of atoms *N* ranging from 2 until 92 (mean: 50.9), see Fig. 1. In doing so, QM conformers were generated using the conformational search workflow implemented in CREST code^56^ (which is short for Conformer-Rotamer Ensemble Sampling Tool) that considers semi-empirical GFN2-xTB^13^ with GBSA implicit solvent model of water^45^. Since vdW interactions have a significant impact on the conformations of large molecules, we have optimized a set of representative conformers using third-order DFTB method^10,57,58^ (or DFTB3) supplemented with a treatment of many-body dispersion (MBD) interactions^59–62^ (see “Methods”). Moreover, to have a better understanding of solvent effects, we have performed these calculations in gas phase and in implicit water described by the GBSA model. For each of the (gas-phase and solvated) optimized conformers, AQM also includes an extensive number (over 40) of global (molecular) and local (atom-in-a-molecule) QM properties computed at a high level of theory that depends on the chemical environment used during the geometry optimization. The majority of QM properties for gas-phase structures were evaluated using non-empirical hybrid DFT with MBD interactions (*i.e*., PBE0+MBD) in conjunction with tightly-converged numeric atom-centered orbitals^63^. In addition, MPB implicit solvent model of water was considered to obtain the properties for solvated structures. Hence, we have two different AQM subsets, namely AQM-gas and AQM-sol, which contain the QM structural and property data of molecules in gas phase and implicit water, respectively. Based on its design, AQM holds the potential to enhance the comprehension of the influence of molecule-solvent and collective dispersion interactions in structure-property and property-property relationships of molecules of pharmaceutically relevant size and composition.

### Key advancements

Our main motivation for proposing AQM as a benchmark dataset is to advance in the development of the next generation of ML models that enable a fast and accurate property estimate and ideation of drug-like molecules synthesized in a chemical environment. In pursuit of this aim, we have ensured that the AQM dataset exhibits the following characteristics,

- The idea of combining the CREST conformational search workflow with the subsequent DFTB3+MBD geometry optimization, both in gas phase and implicit water, has provided us with access to a more extensive and reliable exploration of low- and high-energy (compact/extended) conformers of large molecules. To the best of our knowledge, this procedure has not been considered in previous works. Also, notice that MBD interaction is a key factor in accurately describing and identifying diverse conformations in large molecular complexes, due to their anisotropic shapes.
- The AQM dataset provides a more accurate set of over 40 global (molecular) and local (atom-in-a-molecule) QM properties of gas-phase and solvated structures when compared to already public datasets (see Table 1). These properties can assist in the estimation and comprehension of the impact of molecule-solvent interactions in the structure-property and property-property relationships of large molecules, *e.g*., *via* delta learning approach.
- The property data stored in AQM-gas and AQM-sol can potentially be used to develop more robust QM descriptors for large molecules, enabling fast and accurate calculations of their physicochemical or biological properties. Moreover, such quantum property-based molecular descriptors are complementary to the widely used geometric descriptors and both can be used in synergy for estimating molecular observables measured in experiment.
- The gas-phase and solvated conformations of AQM molecules, along with their highly accurate QM properties make the AQM dataset a valuable resource for in silico assisted methods.

## Methods

### Selection of representative chemistries

We sought to select a set of compounds from the public domain that approximate a typical corporate library including H, C, N, O, F, P, Cl, and S atoms. To this end, we sampled 5000 compounds from ChEMBL^64^ and compared them to the Johnson & Johnson Innovative Medicines corporate database. Compounds with molecular weights over 1200, more than 30 rotatable bonds, a quantitative estimate of drug-likeness (QED) score^65^ under 0.4, and heavy atom count over 200 were removed. Then, we got a reduced ChEMBL set of compounds with similar molecular weights, numbers of rotatable bonds, and fraction of sp3 to the corporate database (see Fig. S1 of the Supplementary Information (SI)). This subset was subjected to diversity selection, followed by manual inspection to remove molecules containing undesirable or unusual chemical substructures. As a result, we initially selected SMILES (which stands for Simplified Molecular Input Line Entry System) of molecular building blocks and typical lead-like compounds as well as a few protein degraders and macrocycles to produce a total of 2, 635 unique molecules with up to 60 non-hydrogen atoms (*N *≤ 116). To have a more extensive sampling of the chemical space described by the selected SMILES, we generated all possible stereoisomers for each structure using the RDKit code—an open source toolkit developed for cheminformatics^66,67^. In our script, we generate unique stereoisomers with the option to perturb each stereocenter while keeping the same atomic connectivity (*i.e*., tautomers are not considered), yielding a larger number of isomers whose stability is later checked *via* quantum mechanical (QM) calculations. Accordingly, the new number of molecular structures considering the stereoisomers is circa 10 k. Initial 3D structures were subsequently generated with RDKit and optimized using the MMFF94 force field^68–72^.

### Generation of molecular conformers

Conformational sampling plays a crucial role in the generation of AQM dataset. We have meticulously explored different conformational search workflows to identify the most effective approach for comprehensively sampling the potential energy surface (PES) and the molecular property space of large drug-like molecules (see “Technical Validation” and Sec. 2 of the SI). In doing so, we opted to use the approach implemented in CREST^56^ code which uses extensive sampling based on the much faster and yet reliable semi-empirical extended tight-binding method (GFN2-xTB)^12,13^ to generate 3D conformations. The semi-empirical energies and structures are thought to be more accurate than classical force fields, accounting for electronic effects, rare functional groups, and bond-breaking/formation of labile bonds^13,46,51,73^. Moreover, the CREST search algorithm is based on metadynamics (MTD), a well-established thermodynamic sampling approach that can efficiently explore the low-energy search space. The collective variables used for the MTD sampling are the atomic root-mean-squared deviation (RMSD) values between the previous structures on the PES of a given molecule^56^. The atomic RMSD values are introduced into the expression of the bias potential, which is used to compute the guiding forces. These forces are responsible for driving the structure further away from previous geometries, providing an extensive exploration of PES. Conformers are thus generated in an iterative manner of MTD and GFN2-xTB optimization, where those geometries are added to the conformer rotamer ensemble (CRE) that overcome certain energy ( > 12.0 kcal/mol) and root-mean-square deviation (Δ*R* > 0.1 Å) thresholds concerning the input structure. The procedure is restarted using the conformer as input if a new conformer has a lower energy than the input structure. The three conformers of lowest energy undergo two normal molecular dynamics (MD) simulations at 400K and 500K, which are used to sample low-energy barrier crossings, such as simple torsional motions. Finally, a genetic Z-matrix crossing algorithm is used and the results are added to the CRE. Then, a normal-type convergence optimization separates the geometries into conformers, rotamers, and duplicates, where duplicates are deleted and conformers and rotamers added to the CRE. Both geometry optimization and conformational search calculations were carried out considering implicit water described by the Generalized Born (GB) model augmented with the hydrophobic solvent accessible surface area term (GBSA)^45^. Finally, we obtained 2, 242, 490 conformers for the initial set of 2, 635 molecules.

Unlike other already public datasets of conformers of large molecules (see Table 1), we have here defined a method to select a set of representative conformers per molecule (*i.e*., per SMILE) instead of considering all conformers generated by CREST. The purpose of using this method is to filter out conformers that are similar in regions of the chemical space defined by the atomic structure, total energy *E*_tot_ and many-body dispersion (MBD) energy *E*_MBD_. We have here considered *E*_MBD_ due to its relevance in the definition of stability rankings in large molecules and molecular crystals^59–62^. Accordingly, our initial step involves determining clusters that consist of conformers exhibiting a root-mean-square deviation (Δ*R*) between their structures of less than 1.5 Å. Δ*R* is computed with the help of DockRMSD tool^74^. After clustering the conformers, we obtained *E*_tot_ and *E*_MBD_ of all conformers per cluster *via* single-point calculations using third-order self-consistent charge density functional tight binding (DFTB3)^10,57,58^ supplemented with a treatment of MBD interactions^59–62^, making use of 3ob parameters^75,76^. Then, we select the conformers with the most distinct values of both energies (*i.e*., *E*_tot_ > 0.24 eV and *E*_MBD_ > 0.048 eV) per cluster, see Fig. 1. This exemplifies a new approach for selecting conformers of large molecules within chemical space, utilizing an in-depth analysis of their electronic properties. To showcase its efficacy, we have considered only 1, 653 molecules ( ≈ 60% of total initial unique molecules) with up to 54 non-hydrogen atoms (*N *≤ 92), reducing the number of conformers for these molecules from 280, 182 to 59, 783.

While this method does indeed yield a more diverse set of conformers, it remains essential to confirm the energetic and mechanical stability of these molecular structures, especially, taking into account the treatment of MBD interactions. To maintain consistency with our earlier publication of the QM7-X dataset for small organic molecules, we have conducted the geometry optimization calculations utilizing the DFTB3+MBD level of theory. Moreover, to construct a dataset that can be used to understand the influence of molecule-solvent interaction on the physicochemical properties of large drug-like molecules, our generation procedure considers the optimization of structures in gas phase and in implicit water described by the GBSA model, as implemented in the DFTB+ code^77^. We have stored the gas-phase and solvated optimized structures into two subsets named AQM-gas and AQM-sol, respectively. These DFTB calculations were performed by interfacing DFTB+ code with the Atomic Simulation Environment (ASE)^78^. Despite the majority of these molecular structures being identified as local minima at the DFTB3+MBD level, both in gas phase and in implicit water, it is worth mentioning that some of them are situated at saddle points on the respective PES.

### Calculation of physicochemical properties

These ≈ 60 k DFTB optimized structures were now utilized for more accurate QM single-point calculations using dispersion-inclusive hybrid DFT. Energies, forces, and several other physicochemical properties (as detailed in Table 2) were calculated at a higher level of theory that varied depending on the chemical environment used in the structure optimization process. Property calculations for AQM-gas molecules were computed using PBE0+MBD^59,79,80^ level, while, for AQM-sol molecules, the modified Poisson-Boltzmann (MPB)^43,81^ model of water was also considered. The MPB model solves the size-modified Poisson-Boltzmann equation for the implicit inclusion of electrolytic solvation effects into DFT calculations. It also includes a model for the well-known Stern layer that separates the diffusing ions from the solvation cavity by introducing non-mean-field ion-solute interactions. For these calculations, we have used the FHI-aims code^82,83^ (version 221103) together with “tight” settings for basis functions and integration grids. Energies were converged to 10^−6^ eV and the accuracy of the forces was set to 10^−4^ eV/Å. The convergence criteria used during self-consistent field (SCF) optimizations were 10^−3^ eV for the sum of eigenvalues and 10^−6^ electrons/Å^3^ for the charge density.

**Table 2:** List of physicochemical properties in the AQM subsets, *i.e*., AQM-gas (without solvent) and AQM-sol (with solvent).

#	Symbol	Property	Unit	Dimension	Type	Level	HDF5 keys	AQM subset
1	*Z*	Atomic numbers	—	*N*	S	—	‘atNUM’	gas/sol
2	*R*	Atomic positions (coordinates)	Å	3*N*	S	TB	‘atXYZ’	gas/sol
3	*I*	Moment of inertia tensor	amu ⋅ Å^2^	9	S	—	‘sMIT’	gas/sol
4	*S*_cav_	Surface area of cavity	a.u.	1	S	P0	‘sCAV’	sol
5	*V*_cav_	Volume of cavity	a.u.	1	S	P0	‘vCAV’	sol
6	*E*_tot_	Total PBE0+MBD energy	eV	1	M,G	P0M	‘ePBE0+MBD’	gas/sol
7	*E*_at_	Atomization energy	eV	1	M,G	P0	‘eAT’	gas/sol
8	*E*_PBE0_	PBE0 energy	eV	1	M,G	P0	‘ePBE0’	gas/sol
9	*E*_MBD_	MBD energy	eV	1	M,G	P0M	‘eMBD’	gas/sol
10	*E*_TS_	TS dispersion energy	eV	1	M,G	P0	‘eTS’	gas/sol
11	*E*_solv_	Free energy in electrolyte	eV	1	M,G	P0	‘eSOLV’	sol
12	*E*_nelec_	Non-electrostatic free energy	eV	1	M,G	P0	‘eNELEC’	sol
13	*E*_nn_	Nuclear-nuclear repulsion energy	eV	1	M,G	—	‘eNN’	gas/sol
14	*E*_kin_	Kinetic energy	eV	1	M,G	P0	‘eKIN’	gas/sol
15	*E*_ne_	Nuclear-electron attraction	eV	1	M,G	P0	‘eNE’	gas/sol
16	*E*_coul_	Classical coulomb energy (el-el)	eV	1	M,G	P0	‘eEE’	gas/sol
17	*E*_xc_	Exchange-correlation energy	eV	1	M,G	P0	‘eXC’	gas/sol
18	*E*_x_	Exchange energy	eV	1	M,G	P0	‘eX’	gas/sol
19	*E*_c_	Correlation energy	eV	1	M,G	P0	‘eC’	gas/sol
20	*E*_xx_	Exact exchange energy	eV	1	M,G	P0	‘eXX’	gas/sol
21	*E*_KS_	Sum of Kohn-Sham eigenvalues	eV	1	M,G	P0	‘eKSE’	gas/sol
22	*ϵ*	Kohn-Sham eigenvalues	eV	*	M,G	P0	‘KSE’	gas/sol
23	*E*_HOMO_	HOMO energy	eV	1	M,G	P0	‘eH’	gas/sol
24	*E*_LUMO_	LUMO energy	eV	1	M,G	P0	‘eL’	gas/sol
25	*E*_gap_	HOMO-LUMO gap	eV	1	M,G	P0	‘HLgap’	gas/sol
26	*D*_s_	Scalar dipole moment	*e* ⋅ Å	1	M,G	P0	‘DIP’	gas/sol
27	*D*	Dipole moment	*e* ⋅ Å	3	M,G	P0	‘vDIP’	gas/sol
28	*Q*_tot_	Total quadrupole moment	*e* ⋅ Å^2^	3	M,G	P0	‘vTQ’	gas/sol
29	*Q*_ion_	Ionic quadrupole moment	*e* ⋅ Å^2^	3	M,G	P0	‘vIQ’	gas/sol
30	*Q*_elec_	Electronic quadrupole moment	*e* ⋅ Å^2^	3	M,G	P0	‘vEQ’	gas/sol
31	*C*_6_	Molecular *C*_6_ coefficient	${E}_{h}\cdot {a}_{0}^{3}$	1	M,R	P0M	‘mC6’	gas/sol
32	*α*_s_	Molecular polarizability (isotropic)	${a}_{0}^{3}$	1	M,R	P0M	‘mPOL’	gas/sol
33	*α*	Molecular polarizability tensor	${a}_{0}^{3}$	9	M,R	P0M	‘mTPOL’	gas/sol
34	*F*_tot_	Total PBE0+MBD atomic forces	eV/Å	3*N*	A,G	P0M	‘totFOR’	gas/sol
35	*F*_PBE0_	PBE0 atomic forces	eV/Å	3*N*	A,G	P0	‘pbe0FOR’	gas/sol
36	*F*_MBD_	MBD atomic forces	eV/Å	3*N*	A,G	P0M	‘vdwFOR’	gas/sol
37	*V*_H_	Hirshfeld volumes	${a}_{0}^{3}$	*N*	A,G	P0	‘hVOL’	gas/sol
38	*V*_ratio_	Hirshfeld ratios	—	*N*	A,G	P0	‘hRAT’	gas/sol
39	*q*_H_	Hirshfeld charges	*e*	*N*	A,G	P0	‘hCHG’	gas/sol
40	*D*_H,s_	Scalar Hirshfeld dipole moments	*e* ⋅ *a*_0_	*N*	A,G	P0	‘hDIP’	gas/sol
41	*D*_H_	Hirshfeld dipole moments	*e* ⋅ *a*_0_	3*N*	A,G	P0	‘hVDIP’	gas/sol
42	$\widetilde{{C}_{6}}$	Atomic *C*_6_ coefficients	${E}_{h}\cdot {a}_{0}^{6}$	*N*	A,R	P0M	‘atC6’	gas/sol
43	${\widetilde{\alpha }}_{{\rm{s}}}$	Atomic polarizabilities (isotropic)	${a}_{0}^{3}$	*N*	A,R	P0M	‘atPOL’	gas/sol
44	*R*_vdW_	vdW radii	*a*_0_	*N*	A,R	P0M	‘vdwR’	gas/sol

Each property is represented by a symbol (with units and dimensions) and can be found in the HDF5 files using the corresponding HDF5 keys. Different property types are distinguished as follows: structural (S), molecular (M), atom-in-a-molecule (A), ground-state (G), and response (R). Different levels of theory are indicated as follows: DFTB3+MBD (TB), PBE0 (P0), and PBE0+MBD (P0M). The P0M label indicates which properties explicitly include dispersion interactions. GBSA and MPB implicit solvent models of water have also been considered for structure optimization and property calculation of the set of molecules in AQM-sol, respectively. *E*_*h*_ and *a*_0_ refer to the atomic units of energy (Hartree) and length (Bohr radius), respectively.

*The number of Kohn-Sham eigenvalues varies for each molecule.

The MBD energies and MBD atomic forces were here computed using the range-separated self-consistent screening (rsSCS) approach^60^, while the atomic *C*_6_ coefficients, isotropic atomic polarizabilities, molecular *C*_6_ coefficients and molecular polarizabilities (both isotropic and tensor) were obtained *via* the SCS approach^59^. Hirshfeld ratios correspond to the Hirshfeld volumes divided by the free atom volumes. The TS dispersion energy refers to the pairwise Tkatchenko-Scheffler (TS) dispersion energy in conjunction with the PBE0 functional^49^. The vdW radii were also obtained using the SCS approach *via*${R}_{{\rm{vdW}}}={\left({\alpha }^{{\rm{SCS}}}/{\alpha }^{{\rm{TS}}}\right)}^{1/3}{R}_{{\rm{vdW}}}^{{\rm{TS}}}$, where *α*^TS^ and ${R}_{{\rm{vdW}}}^{{\rm{TS}}}$ are the atomic polarizability and vdW radius computed according to the TS scheme, respectively. Atomization energies were obtained by subtracting the atomic PBE0 energies from the PBE0 total energy of each gas-phase and solvated molecular conformation (see Table S1 of the SI). The exact exchange energy is the amount of exact (or Hartree-Fock) exchange that has been admixed into the exchange-correlation energy.

## Data Records

The AQM dataset is provided in two HDF5 files in a ZENODO.ORG data repository^84^. The QM structural and property data of the 59, 783 conformations corresponding to 1, 653 molecules in both gas phase and implicit water were stored in the AQM-gas.hdf5 and AQM-sol.hdf5 files, respectively. Additionally, we have uploaded the AQM-initial.hdf5 file which only contains the structural data of the 2, 242, 490 conformations corresponding to the initial set of 2, 635 molecules (obtained by using CREST code). One can also find there a README file with technical usage details and an example of how to access the information stored in AQM (see readAQM.py file).

### HDF5 file format

Independent of the AQM subset, the information for each molecular structure is stored in a Python dictionary (dict) type containing all relevant properties and recorded in *groups* in HDF5 file format^30^. HDF5 keys to access the atomic numbers, atomic positions (coordinates), and physicochemical properties in each dictionary are provided in Table 2. The dimension of each array depends on the number of atoms *N* and the required property, *e.g*., for a methane (CH_4_) molecule, ’atNUM’ is a 1D array of *N* = 5 elements ([6, 1, 1, 1, 1]) while ’atXYZ’ is a 2D array comprised of *N* = 5 rows and three columns (*x*, *y*, *z* coordinates). All structures are labeled as *Geom-mr-ct*, where *r* enumerates the SMILE strings and *t* the considered conformer. Note that the indices *t* used in the AQM dataset reflect the order in which a given structure was generated and do not correspond to sorted xTB/DFTB (or DFT) total energies.

## Technical Validation

A significant challenge in simulating the physicochemical properties of large drug-like molecules lies in the fact that, in experiments, their conformations and electronic structures are influenced by interactions with the surrounding solvent. However, the standard approach in contemporary QM simulations involves running them in gas phase, without accounting for molecule-solvent interactions. Unlike another recently published dataset of large molecules (see Table 1), AQM dataset considers the molecule-solvent interactions as well as a treatment of van der Waals (vdW) interactions in its generation procedure—two important physical and chemical effects in determining structural conformations and stability rankings of molecules of pharmaceutically relevant size. As mentioned above, the AQM comprises the structural and electronic data of 59, 783 gas-phase and solvated (low-and high-energy) conformers of 1, 653 molecules with up to 54 non-hydrogen atoms (*N *≤ 92), including C, N, O, F, P, S and Cl. The structures of solvated conformers were obtained using DFTB3+MBD method supplemented with the GBSA implicit model of water. This model has been successfully used in the study of free solvation energies of neutral/ionic molecules^45^ and the folding of short peptides^85^. Whereas, the level of theory selected to compute the QM properties per conformer was PBE0+MBD supplemented with the MPB implicit model of water. The MPB model has been shown to provide a more accurate description in the study of diverse electrochemical reactions^86–89^. In all calculations, many-body dispersion (MBD) interactions have been included to deal with long-range interactions that are not adequately represented by the baseline level of theory. These advanced theoretical models have thus generated a more accurate collection of molecular and atom-in-a-molecule (as well as ground state and response) QM properties of conformers in implicit water, which are stored in AQM-sol. Moreover, when integrated with the property information in AQM-gas, these data can assist in fine-tuning ML models for the precise estimation of electronic properties of solvated molecules, *e.g*., *via* a delta learning approach.

**Fig. 2:**
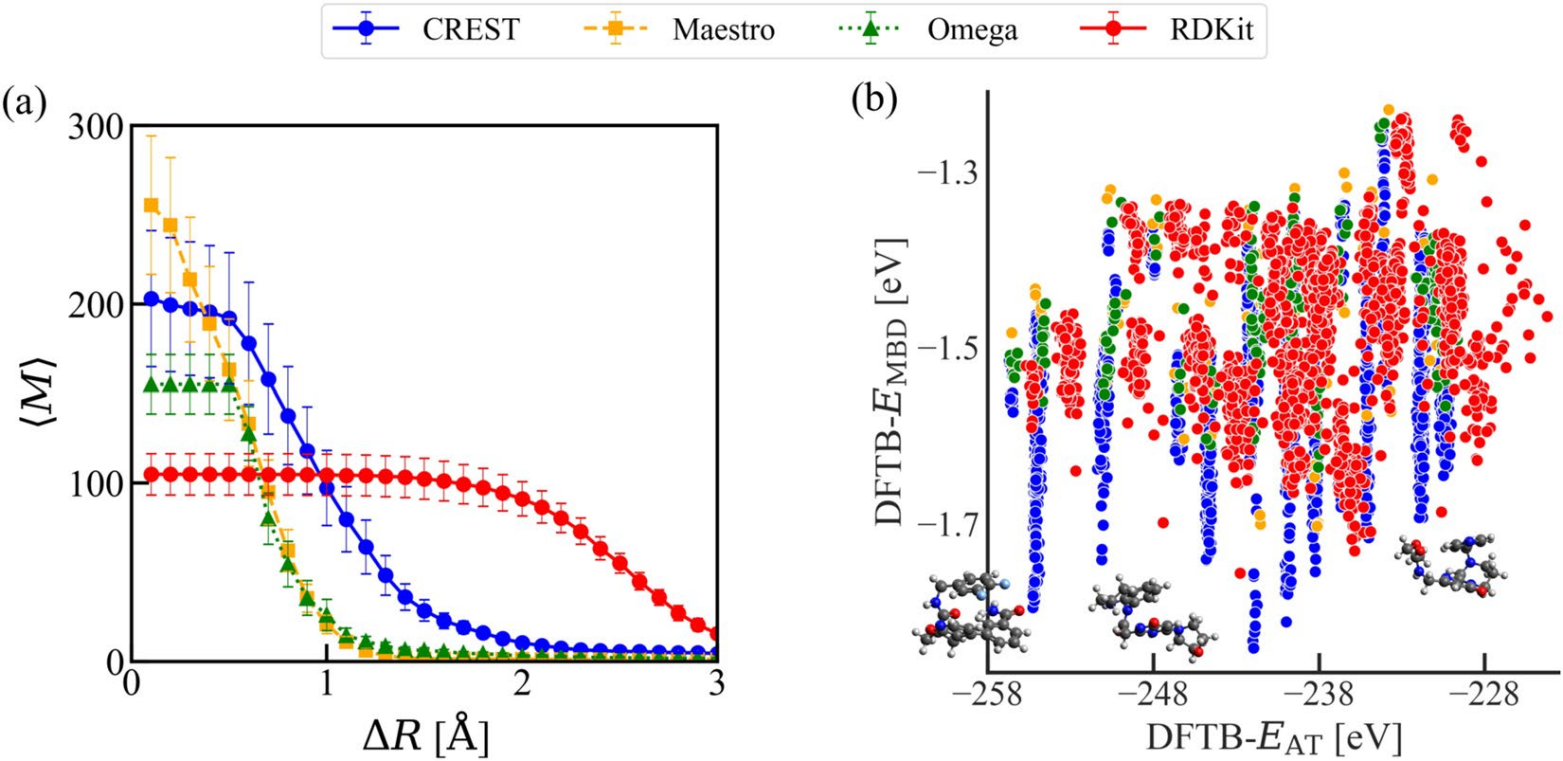
Comparison of the performance of conformational search workflows. (a) Variation of the averaged number of clusters ⟨*M*⟩ as a function of the root-mean-square error Δ*R* between conformers generated by the four conformational search workflows examined in this work (see legend on top). The average was taken over 18 randomly selected compositions with approximately *N* = 50 atoms. (b) Two-dimensional property space defined by *E*_AT_ and *E*_MBD_ of the set of representative conformers obtained after applying the criteria defined in “Methods”. The energy calculations were performed using DFTB3+MBD method. Examples of structures only generated by CREST code are inserted in the graph.

An essential step in the generation of AQM dataset involved the thoughtful selection of the conformational search workflow. Here, we exhaustively analyzed the sampling method implemented in four different codes: CREST^56^, Maestro, Omega^90^ and RDKit^66,91^. The last three codes are of standard use in cheminformatics, primarily relying on stochastic algorithms for their application. They explore the conformational space of molecules very sparsely through a combination of pre-defined distances and stochastic samples^92^ and can miss many low-energy conformations. Moreover, in most standalone applications, conformer energies are typically computed using classical force fields without the incorporation of solvent models, which made these values rather inaccurate^93^ (for more details of these methods see Sec. 2 of the SI). On the contrary, CREST code utilizes a robust sampling strategy, leveraging the semi-empirical extended tight-binding method (GFN2-xTB)^13^, supplemented by the GBSA implicit solvent model of water, in order to generate more reliable 3D conformations compared to those obtained using classical force fields (see “Methods”). The conformational search workflow implemented in CREST provides access to a more extensive exploration of low- and high-energy conformers, generating molecular structures inaccessible by distance geometry methods (see Fig. 2)^46,51,73^.

To gain a better understanding of the influence of these sampling methods on the conformational search of large drug-like molecules, we analyzed the structural and energetic data of conformers corresponding to 18 randomly selected compositions, each containing approximately *N* = 50 atoms. Fig. 2(a) displays the output for the variation of the averaged number of clusters, denoted as ⟨*M*⟩, as a function of the root-mean-square deviation, Δ*R*, among conformers that constitute a cluster. This calculation was first done per molecule and then averaged over the 18 cases. This showed that more diverse conformers become part of the same cluster when Δ*R* increases, resulting in a reduction of the number of distinct clusters. The decrease in ⟨*M*⟩ for Maestro and Omega is faster compared to CREST, which may indicate that conformational search workflows based on distance geometry methods are insufficient for probing the PES of more flexible molecules. However, thanks to the random ensemble option for conformer generation, RDKit produces a larger ⟨*M*⟩ than CREST for Δ*R* > 1.0 Å. To examine this result further, we compute *E*_AT_ and *E*_MBD_ for a set of representative conformers per molecule (see selection criterion in “Methods”) using DFTB3+MBD level of theory. Notice that the resulting total number of conformers depends on the code employed for their generation, *i.e*., CREST → 3747 conformers, Maestro → 100 conformers, Omega → 204 conformers and RDKit → 1872 conformers. Thus, we have found that, compared to other methods, the conformers generated by CREST code show a more organized coverage of the energetic space defined by DFTB-*E*_AT_ and DFTB-*E*_MBD_ (see the well-defined cluster in Fig. 2(b)). Moreover, the larger coverage of DFTB-*E*_MBD_ values is a clear indicator that CREST sampling method can generate conformations of increased complexity, characterized by a more folded structure and a stronger dispersion interaction, as illustrated with the inserted structures in Fig. 2(b). This underscores the relevance of taking vdW interactions into account when generating and identifying conformers of large drug-like molecules. Similarly, this provides compelling evidence of the efficient conformational search workflow implemented in CREST code, which improves coverage of both conformational and property molecular space. It is noteworthy that the calculations executed by the CREST code incurred higher computational expenses compared to those carried out by chemoinformatics codes.

**Fig. 3:**
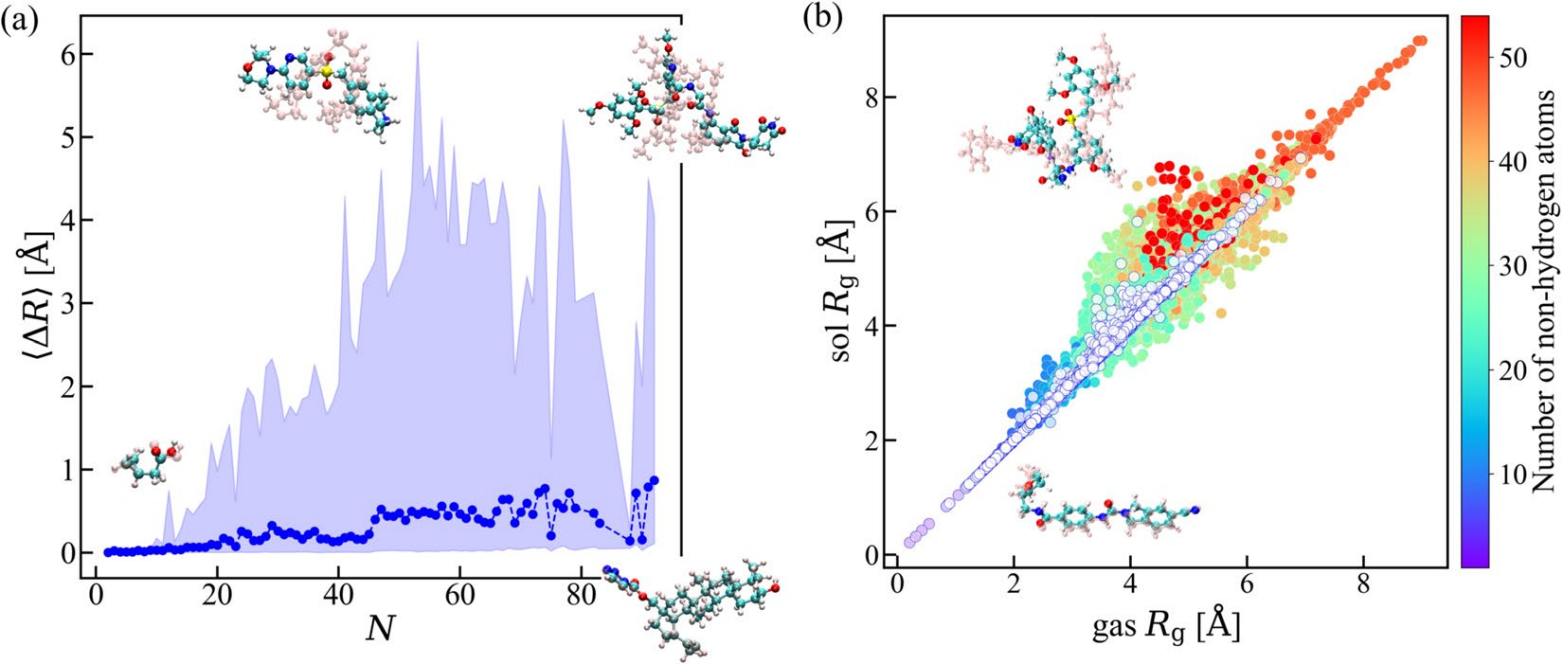
Effect of solvation on the atomic structures. **(a)** Size dependence of averaged root-mean-square deviation ⟨Δ*R*⟩ between geometries optimized using DFTB3+MBD method in gas phase and in implicit water described by the GBSA model. We also show the range of Δ*R* values spanned at different *N* (blue shadow). **(b)** Correlation plot between the gyration radius *R*_*g*_ of gas-phase and solvated conformations. Datapoints are colored with respect to the number of non-hydrogen atoms. To perform these analyses, we have considered the 59, 783 low-and high-energy conformers contained in AQM-gas and AQM-sol. In each panel, select conformations were inserted to highlight the effect of solvent on the molecular structure. Solvated structures are represented with pink balls.

After generating all conformers and selecting the representative conformers for each molecule, we proceed to optimize the molecular structures. This optimization is carried out using the DFTB3+MBD level of theory in the gas phase as well as in implicit water described by the GBSA model. Once this optimization step is complete, we obtain the final set of molecular structures for AQM-gas (gas phase) and AQM-sol (implicit water) subsets. To quantitatively analyze the impact of molecule-solvent interaction on the structure of AQM molecules, we have computed Δ*R* between the molecular structures stored in AQM-gas and AQM-sol. Indeed, Fig. 3(a) displays the size dependence of the averaged Δ*R* (blue dots) together with the total range of Δ*R* values spanned at different *N* (blue shadow). The results show that the structures of small molecules (*N* ≤ 20 atoms) present ⟨Δ*R*⟩ < 0.1 Å, *i.e*., they are minimally affected by the interaction with the solvent. In contrast, when dealing with molecules with *N* > 40 atoms, solvent effects become significantly more pronounced, and, as a result, we observe greater deviations in the Δ*R* values (> 2.0 Å). A similar effect can be observed when comparing the gyration radius *R*_*g*_ for gas-phase and solvated molecular structures, see Fig. 3(b). Notice that there also are large compounds (*N* ≈ 90) characterized by extensively constrained structures, which remain unaffected by the interaction with implicit water. These findings hold significant relevance in the context of advancing QM-based pipelines for the creation of datasets of molecules of pharmaceutical relevant size. Particularly in cases where the research objectives encompass the generation of non-equilibrium structures for training ML force fields since the PES of these molecules will be largely modified by solvent effects—a phenomenon that can be inferred from the outcomes of the geometry optimization process.

The AQM dataset considers an extensive array of more than 40 distinct molecular (global) and atom-in-a-molecule (local) QM properties, which were computed to gain insights into the effect of molecule-solvent interactions on structure-property and property-property relationships of large molecules. In Table 2, we list the properties of gas-phase and solvated molecules, derived from QM calculations performed at the PBE0+MBD level (AQM-gas) and further enhanced with the MPB implicit solvent model of water (AQM-sol), respectively. PBE0+MBD has been chosen as our baseline level of theory for property calculations due to its well-established accuracy and reliability, demonstrated in the description of intramolecular degrees of freedom as well as intermolecular interactions in organic molecular dimers, supramolecular complexes, and molecular crystals^59,79,80,94–97^. The use of these DFT methods also provides interesting insights into the effect of molecule-solvent interactions on the potential energy surface of molecules, highlighting the importance of the dataset generation procedure. In Fig. S2 of the SI, one can see that the energy range and the energetic ranking of molecules in AQM-gas and AQM-sol are different, but further analysis is required. We therefore consider that our QM calculations are suitable to validate the quality of future research utilizing the AQM dataset.

**Fig. 4:**
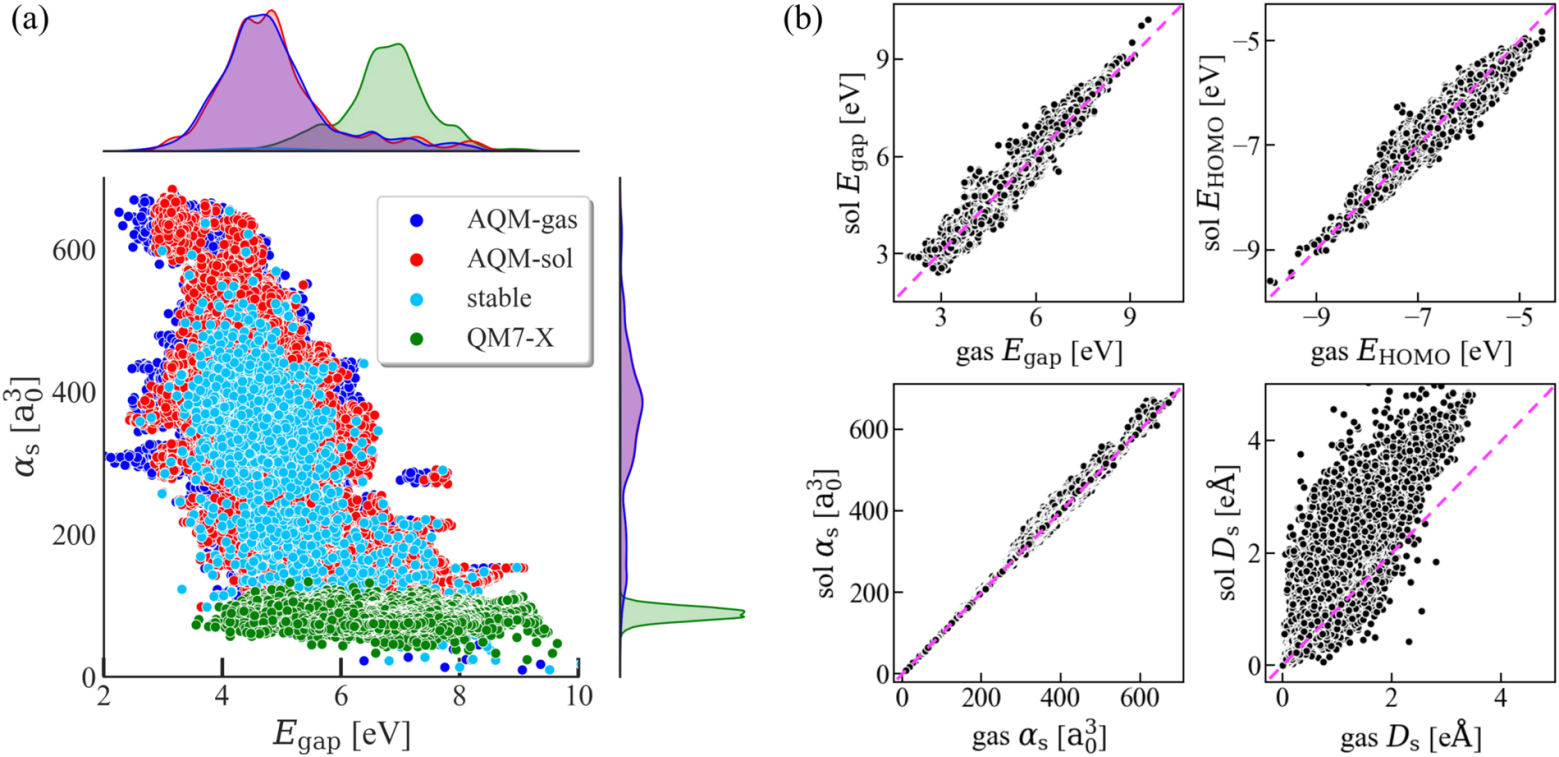
Influence of solvent in select global (molecular) QM properties considered in the AQM dataset. **(a)** 2D projections of the high-dimensional AQM property space defined by isotropic molecular polarizability *α*_s_ and HOMO-LUMO gap *E*_gap_, *i.e*., $\left({\alpha }_{{\rm{s}}},{E}_{{\rm{gap}}}\right)$-space. We show the property values of 59, 783 low-and high-energy conformers contained in AQM-gas (blue circles) and in AQM-sol (red circles) as well as the corresponding values for the most stable conformer of the 1, 653 unique molecules in AQM-sol (cyan circles). For comparison, we also present the values obtained for QM7-X equilibrium molecules (green circles). The frequency plot (distribution) associated with each property and subset of molecules is plotted on the top and right-side panels of the graph. **(b)** Correlation plots between the property values corresponding to 59, 783 gas-phase and solvated structures. Here, we have considered extensive and intensive QM properties such as HOMO-LUMO gap *E*_gap_, HOMO energy *E*_HOMO_, isotropic molecular polarizability *α*_s_, and scalar dipole moment *D*_s_.

To understand the relevance of accessing QM data for solvated molecules, we first discuss the influence of implicit water on extensive and intensive molecular QM properties. In doing so, as an illustrative example, we have analyzed the 2D property space defined by two contrasting properties^98,99^ such as the isotropic molecular polarizability *α* and the HOMO-LUMO gap *E*_gap_ (*i.e*., $\left(\alpha ,{E}_{{\rm{gap}}}\right)$-space) for the 59, 783 conformations in AQM-gas and AQM-sol as well as the set of most stable conformer per molecule in AQM-sol (only 1, 653 conformations), see Fig. 4(a). For comparison, the values corresponding to QM7-X equilibrium molecules are also plotted (green circles). Our findings reveal that AQM molecules exhibit a significantly broader coverage of the *α* range, surpassing QM7-X molecules by a factor of 6. This expanded coverage is attributed to the inherently extensive character of *α*. Whereas, *E*_gap_ range now covers molecules with circa 2.5 eV of energy gap, and the mean value for the entire dataset reduced from 7.0 eV to 4.5 eV, see distribution plots on the top panel of Fig. 4(a). The slight differences between the $\left(\alpha ,{E}_{{\rm{gap}}}\right)$-space covered by AQM-gas and AQM-sol may be attributed to compensation between the pronounced fluctuations in *α*, which are predominantly observed in molecules with $\alpha  > 300\,{a}_{0}^{3}$, and the more sensitive behavior of *E*_gap_ to the presence of implicit water, as displayed in the correlation plots in Fig. 4(b). Accordingly, these findings could carry crucial implications in the “freedom of design” when searching for large drug-like molecules with targeted $\left(\alpha ,{E}_{{\rm{gap}}}\right)$ values^98–100^. Notice that the conformational sampling per molecule largely improved the coverage of both properties, connecting isolated regions associated with a single molecular structure with specific size and chemical composition. Fig. 4(b) also shows the correlation plots between HOMO energy *E*_HOMO_ and total dipole moment *D*_*s*_ of the 59, 783 conformers contained in AQM-gas and AQM-sol. Thus, it becomes evident that intensive properties are more sensitive to the incorporation of molecule-solvent interactions in the QM calculations when contrasted with extensive properties.

**Fig. 5:**
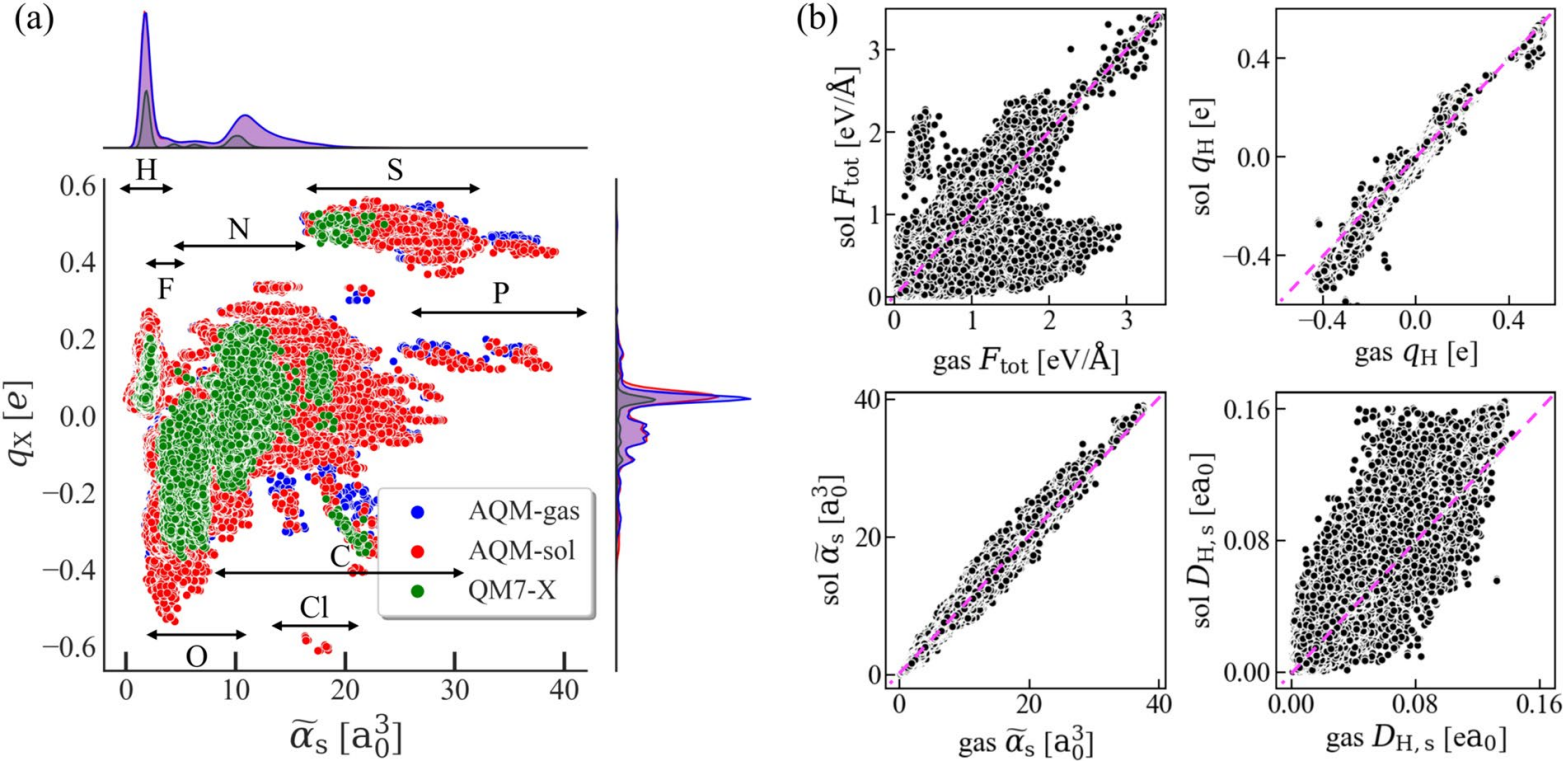
Influence of solvent in select local (atom-in-a-molecule) QM properties considered in the AQM dataset. **(a)** 2D projections of the high-dimensional AQM property space defined by Hirshfeld charges *q*_H_ and atomic polarizabilities ${\widetilde{\alpha }}_{{\rm{s}}}$, *i.e*., $\left({q}_{{\rm{H}}},{\widetilde{\alpha }}_{{\rm{s}}}\right)$-space. We show the property values of 59, 783 low-and high-energy conformers contained in AQM-gas (blue circles) and AQM-sol (red circles). For comparison, we also present the values obtained for QM7-X equilibrium molecules (green circles). The arrows in the graph highlight the region covered per local chemical environment corresponding to each atom type (X). The frequency plot (distribution) associated with each property and subset of molecules is plotted on the top and right-side panels of the graph. **(b)** Correlation plots between the property values corresponding to 59, 783 gas-phase and solvated structures. Here, we have considered local QM properties such as total atomic forces *F*_tot_, Hirshfeld charges *q*_H_, atomic polarizabilities ${\widetilde{\alpha }}_{{\rm{s}}}$, and atomic Hirshfeld dipole moment *D*_H,s_.

On the other hand, atom-in-a-molecule QM properties can provide important insight into the distinct chemical environments within large drug-like molecules. To illustrate this, Fig. 5(a) shows the 2D property space defined by Hirshfeld charges *q*_H_ and atomic polarizabilities ${\widetilde{\alpha }}_{{\rm{s}}}$ (*i.e*., $\left({q}_{{\rm{H}}},{\widetilde{\alpha }}_{{\rm{s}}}\right)$-space) for the 59, 783 conformations in AQM-gas and AQM-sol. For comparison, the values corresponding to QM7-X equilibrium molecules are also plotted (green circles). Fig. 5(a) shows the existence of well-defined clusters that are mostly related to a specific atom type. The slight overlap between these clusters is a clear example of the need to develop more robust geometric and electronic descriptors capable of effectively representing intricate chemical environments in large drug-like molecules for ML applications. Furthermore, our calculations have revealed that implicit solvation has a pronounced influence on *q*_H_ values, particularly for heavier atoms such as P, S, and Cl—relevant atoms in the design of pharmaceutical compounds as well as in the determination of their physicochemical and biological properties. Certainly, the molecule-solvent interaction has a stronger effect on the local properties compared to global ones, as illustrated by the correlation plots in Fig. 5(b). This becomes more evident by observing the atomic forces *F*_tot_, where the significant variations in values can strongly affect the accuracy of ML force fields when applied to run the dynamics of large molecules.

**Fig. 6:**
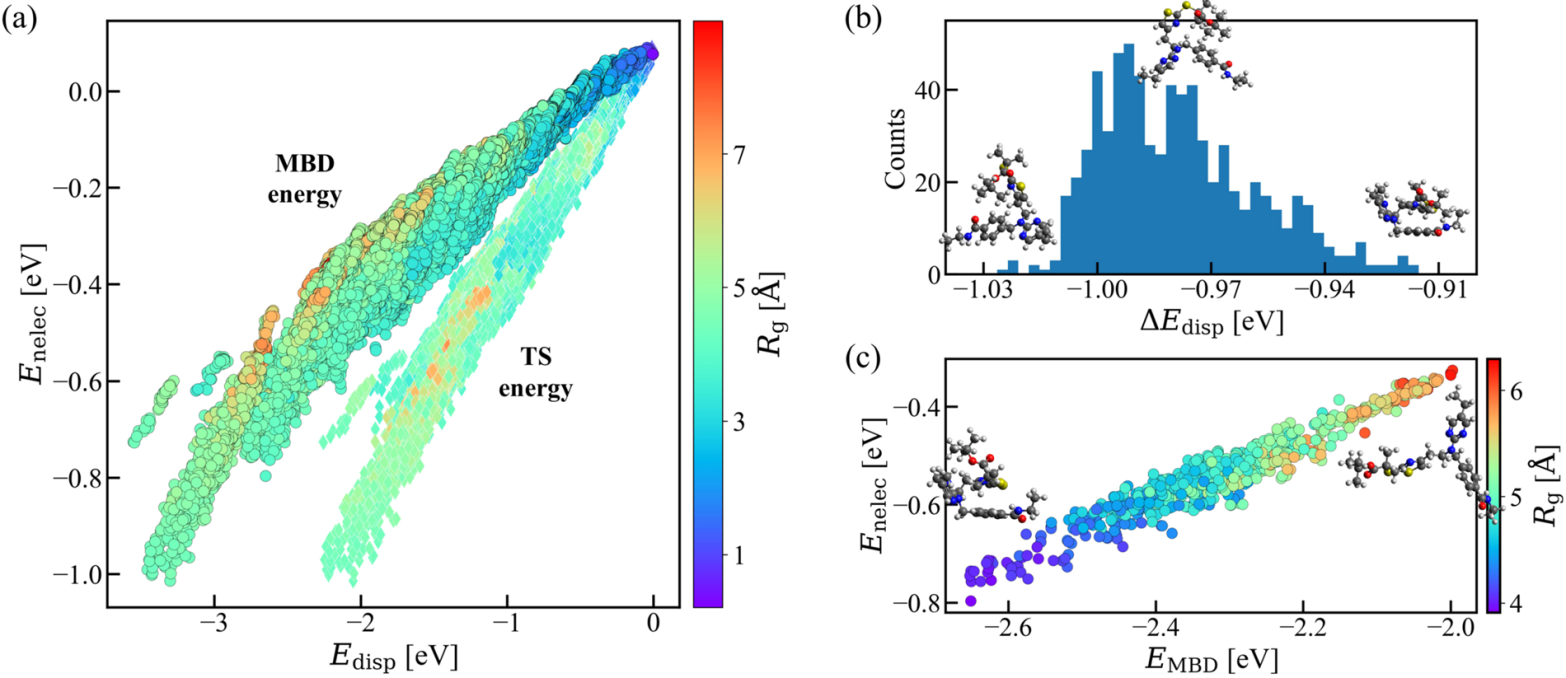
Relevance of dispersion and molecule-solvent interactions in drug-like molecules. **(a)** Variation of the non-electrostatic free energy *E*_nelec_ as a function of the dispersion energy *E*_disp_ for all solvated structures in AQM-sol. We have considered two different dispersion models in this analysis: Many-body dispersion (MBD) and Tkatchenko-Scheffler (TS). **(b)** Frequency plot of the difference between MBD and TS energies (*i.e*., Δ*E*_disp_ = *E*_*MBD*_ − *E*_*TS*_) for the 720 low-and high-energy conformers of the molecule C_29_H_39_N_5_O_3_S_2_ (ID in dataset: 2070). **(c)** Variation of *E*_nelec_ as a function of *E*_MBD_ for all conformers of the molecule C_29_H_39_N_5_O_3_S_2_. The color code in panels (a) and (c) is with respect to the gyration radius *R*_g_ of each solvated structure. We have inserted select conformations in panels (b) and (c) to highlight the effect on the molecular structure of reducing or increasing the magnitude of both properties.

Up to now, we have been focused on the impact of considering molecule-solvent interaction when computing molecular/atom-in-a-molecule QM properties of AQM molecules. However, the data of the non-electrostatic part of solvation energy due to molecule-solvent interaction *E*_nelec_ in conformers contained in AQM-sol can also be crucial for having a better understanding of the solvation effect on structure-property and property-property relationships of large molecules. As an example, Fig. 6(a) shows the correlation plot between *E*_nelec_ and dispersion interaction energy *E*_disp_ calculated using two well-established methods: many-body dispersion (MBD) and Tkatchenko-Scheffler (TS). The datapoints are colored according to the gyration radius *R*_g_ of each solvated structure. The high degree of correlation between these properties underscores the importance of considering both molecule-solvent and dispersion interactions when investigating large molecules, as we did to generate AQM dataset. Moreover, the growing difference in energies obtained by MBD and TS methods, particularly as the system size increases, highlights the significant influence of many-body interactions in the energetic description of these compounds. To further elucidate the role of both interactions in the generation of AQM, we have selected the molecule C_29_H_39_N_5_O_3_S_2_ (*N* = 78 atoms, ID in dataset: 2070) with 720 conformers and then examined their respective energy values. These conformers show a difference in dispersion energies Δ*E*_disp_ of up to ≈1.0 eV, where the smallest Δ*E*_disp_ values correspond to more compact molecular structures while the largest Δ*E*_disp_ values are observed for more extended ones (see Fig. 6(b)). Besides presenting a size dependence, the data plotted in Fig. 6(c) demonstrate that, similar to *E*_MBD_, *E*_nelec_ also depends on the structural conformation of molecules. These findings show the significance of both interactions in the generation of a robust QM dataset comprising large and more flexible molecules that bear pharmaceutical relevance.

In summary, we demonstrated that the extensive structural and property data contained in AQM dataset hold the potential to enhance the understanding of how molecular-solvent interactions influence both structure-property and property-property relationships of large drug-like molecules. As such, AQM may in some cases be employed as a benchmark dataset for direct/delta learning and generative methods, estimating the properties of pharmaceutical compounds in solution from their gas-phase counterparts.

### Supplementary information

SUPPLEMENTARY INFORMATION

## Data Availability

The initial structure generation was carried out using RDKit 2020.09.5^66,67^. Further structure optimization and the creation of conformers were performed by utilizing CREST^13,56^ and DFTB+^10,77^ codes together with ASE^78^. Note that all necessary features regarding the utilized DFTB3+MBD (with and without GBSA implicit solvent) approach are available in the current DFTB+ version^11^. All DFT calculations were carried out using FHI-aims^82^ (version 221103). Additional conformer generation experiments were performed with RDKit 2020.09.5, OpenEye’s Omega 4.0.0.4^90^ and Schrodinger’s Maestro suite v2020-4 (see SI for detailed procedures).
