# Supplementary material for: Dataset for quantum-mechanical exploration of conformers and solvent effects in large drug-like molecules
Source: Sci Data. 2024 Jul 7;11:742. doi: 10.1038/s41597-024-03521-8 (PMC11228031; doi:10.1038/s41597-024-03521-8)
Supplement: Supplementary file 1 — SUPPLEMENTARY INFORMATION [file 41597_2024_3521_MOESM1_ESM.pdf]

Supplementary Information

Dataset for quantum-mechanical exploration of conformers and solvent effects in large drug-like molecules

Leonardo Medrano Sandonas,[*1,2] **Dries Van Rompaey,**[*3] **Alessio Fallani,**[1,3] **Mathias Hilfiker,**[1] **David Hahn,**[4] **Laura Perez-Benito,**[4] **Jonas Verhoeven,**[3] **Gary Tresadern,**[4] **Joerg Kurt Wegner,**[3] **Hugo Ceulemans,**[3] **and Alexandre Tkatchenko**[*1]

[1] *Physics and Materials Science Research Unit, University of Luxembourg, L-1511 Luxembourg City, Luxembourg.*
[2] *Institute for Materials Science and Max Bergmann Center of Biomaterials, TU Dresden, 01062 Dresden, Germany.*
[3] *Drug Discovery Data Sciences, Janssen Pharmaceutica NV, Turnhoutseweg 30, 2340 Beerse, Belgium.*
[4] *Computational Chemistry, Janssen Pharmaceutica NV, Turnhoutseweg 30, 2340 Beerse, Belgium.*

[*] Corresponding authors: Leonardo Medrano Sandonas (`leonardo.medrano@tu-dresden.de`), Dries Van Rompaey (`dvanrom1@its.jnj.com`), Alexandre Tkatchenko (`alexandre.tkatchenko@uni.lu`)

1 Additional properties of AQM molecules

Figure S1 shows the selected molecular properties of the chemical compounds contained in the Aquamarine dataset. These compounds have been selected to approximate the composition of typical corporate databases with regard to typical molecular descriptors such as molecular weight, number of hydrogen bond donors/acceptors, and rotatable bonds. For all properties examined herein, the compounds mostly adhere to the recommendations outlined in Lipinsky's rule of five[1] (no more than one violation of the following: MW<500, cLogP<5, HBA<5, HBD<10) or Veber's rule[2] (rotatable bonds<10, TPSA<140), both of which are widely used guidelines for to increase the chances of oral bioavailability as well as guidelines to enhance overall drug-likeness.

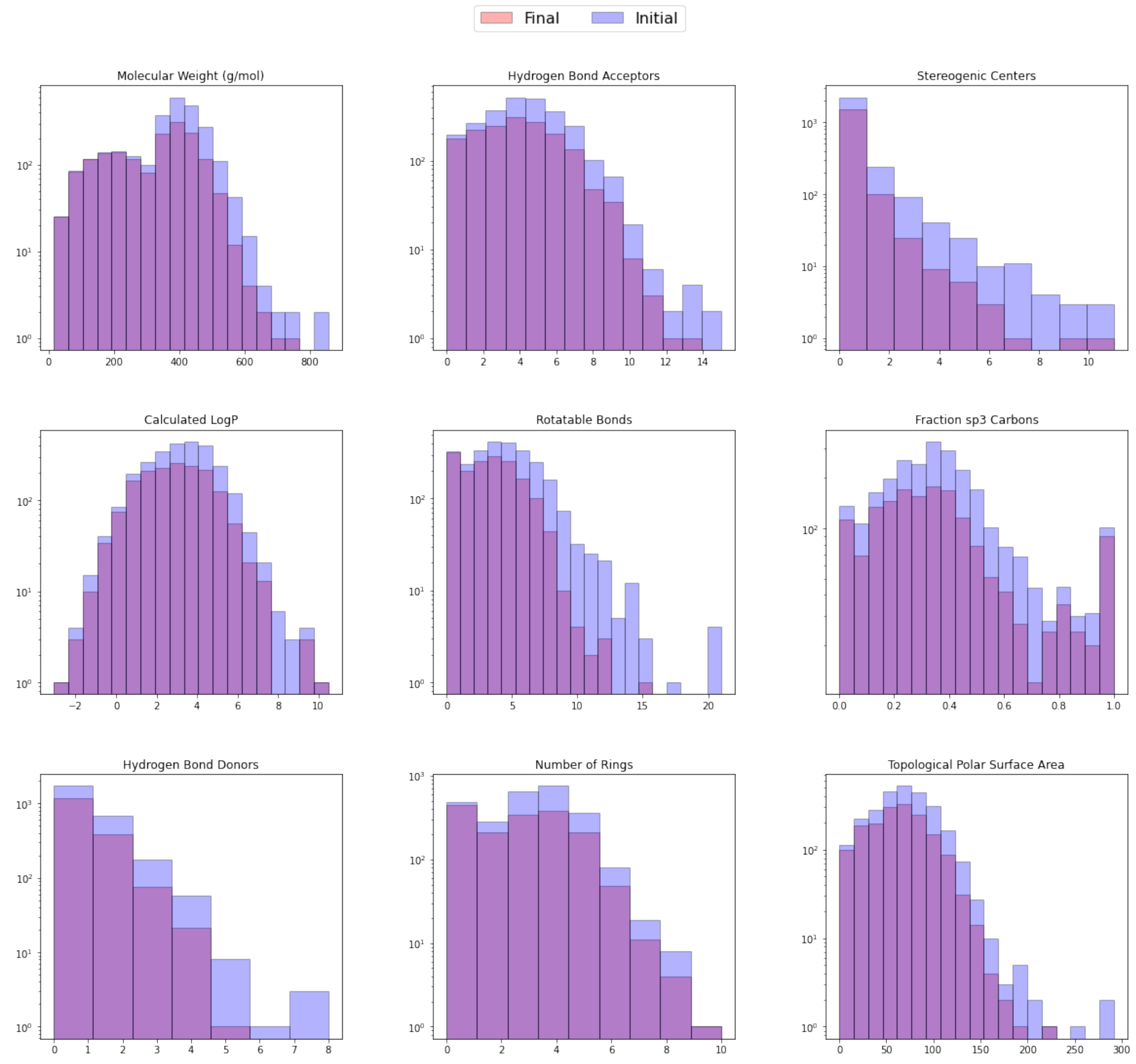

Fig. S1 Frequency plots of the selected properties of molecules contained in the initial and final sets of Aquamarine dataset.

2 Conformational search workflows

In addition to the CREST sampling outlined in the main text, conformers were generated with three chem-informatics packages. OpenEye Omega 4.0.0.4[3] was used in the "classic" mode to generate conformers based on the exhaustive, knowledge-based enumeration of rotamer states of a compound's fragments. The "classic" mode filters out conformers with a strain energy higher than 10 kcal/mol as calculated with a modified version MMFF94[4] without Coulomb terms. After deduplication using a heavy atom root-mean-square Cartesian distance cutoff of 0.5 Å, up to 200 lowest energy conformers were kept. We additionally generated conformers using Rdkit's 2020.09.5[5] ETKDG v3 conformer generator[6]. Two hundred conformers were generated per molecule using random coordinate generation followed by minimization in the distance field. Duplicate structures were pruned using a heavy atom root-mean-square distance of 0.5 Å. Finally, conformers were generated with Schrodinger's Maestro suite. The ligand molecules were prepared using the LigPrep tool. All default settings were used. The ligands were parametrized for use with the OPLS forcefield using the tools available in Maestro. The conformational search was performed using Macromodel module available in Maestro v2020-4. We chose the Mixed torsional/Low mode sampling, using the OPLSv3e forcefield, solvent and other parameters were set at default. Conformers within 10 kcal/mol from the global minimum were collected for output. Duplicate structures were pruned using a heavy atom root-mean-square distance of 0.5 Å.

3 Atomic energies

Chemical element	Energy [eV]	
	AQM-gas	AQM-sol
H	-13.64140416	-13.64332105
C	-1027.60791501	-1027.61074626
N	-1484.27481909	-1484.27621709
O	-2039.75030551	-2039.75167568
F	-2710.54734321	-2710.54812971
P	-9283.01120605	-9283.01586200
S	-10828.72289453	-10828.72622208
Cl	-12516.46004579	-12516.46233936

Table S1 Atomic energies used to compute atomization energies for gas-phase and solvated conformations. These values were obtained at the PBE0 level using FHI-aims. Each atom was treated with the proper spin state for the neutral species.

4 Validation of energetic levels of conformers in AQM-gas and AQM-sol

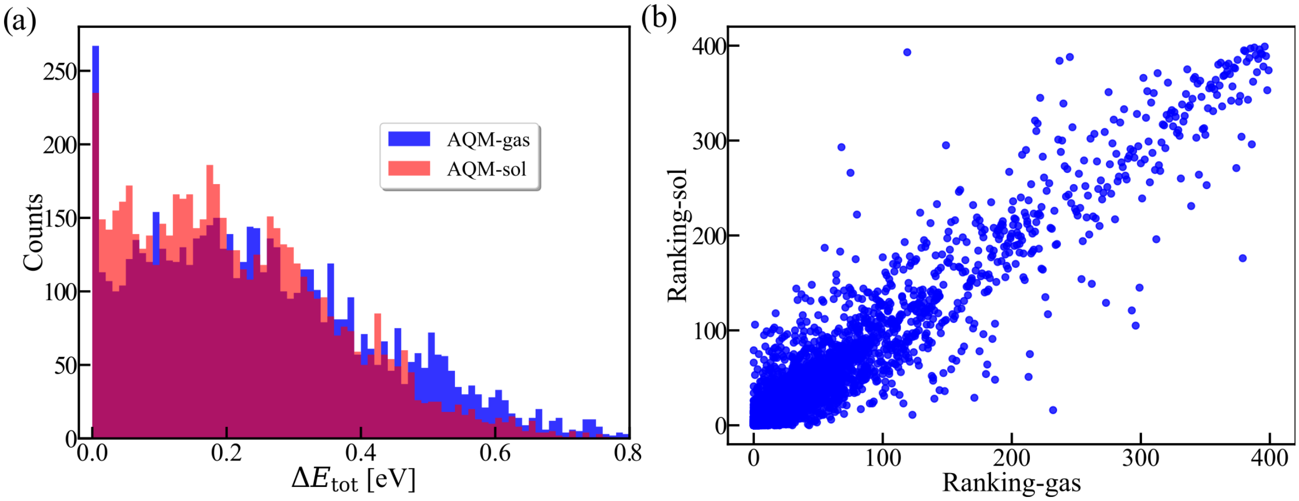

Fig. S2 Validation of energetic levels of conformers in AQM dataset. (a) Frequency plot of the difference ΔE_{tot} between the energy of the local minima and the energy of the global minimum for all selected molecules. (b0) Correlation plot comparing the energetic rankings for the conformers per selected molecule in the gas phase (gas) and implicit solvent (sol). For both calculations, we have analyzed the DFT energy spectrum of 154 molecules with a ranging number of atoms from 50 to 52, which accounts for a total of 6,217 conformers.

References

[1] Lipinski, C. A., Lombardo, F., Dominy, B. W. & Feeney, P. J. Experimental and computational approaches to estimate solubility and permeability in drug discovery and development settings. *Advanced drug delivery reviews* **64**, 4–17 (2012).

[2] Veber, D. F. *et al.* Molecular properties that influence the oral bioavailability of drug candidates. *Journal of medicinal chemistry* **45**, 2615–2623 (2002).

[3] Hawkins, P. C., Skillman, A. G., Warren, G. L., Ellingson, B. A. & Stahl, M. T. Conformer generation with omega: algorithm and validation using high quality structures from the protein databank and cambridge structural database. *Journal of Chemical Information and Modeling* **50**, 572–584 (2010).

[4] Halgren, T. A. Merck molecular force field. i. basis, form, scope, parameterization, and performance of mmff94. *Journal of computational chemistry* **17**, 490–519 (1996).

[5] Landrum, G. *et al.* RDKit: Open-source cheminformatics. https://www.rdkit.org. (2020).

[6] Wang, S., Witek, J., Landrum, G. A. & Riniker, S. Improving conformer generation for small rings and macrocycles based on distance geometry and experimental torsional-angle preferences. *Journal of Chemical Information and Modeling* **60**, 2044–2058 (2020).
